# Surgical management of anterior mediastinal tumors of thyroid origin: a comprehensive analysis of approaches, techniques, and outcomes

**DOI:** 10.1186/s13019-024-02831-7

**Published:** 2024-06-21

**Authors:** Georgi Yankov, Magdalena Alexieva, Yordanka Yamakova, Dimitar Kyuchukov, Evgeni Mekov

**Affiliations:** 1 Department of Respiratory Diseases, Medical University - Sofia, UMHAT ‘St. Ivan Rilski’15, ‘Acad. Ivan Geshov’ Blvd, 1431 Sofia, Bulgaria; 2 Department of Anesthesiology and Intensive Care, Medical University - Sofia, Sofia, Bulgaria; 3 Cardiovascular Surgery Department, Medical University - Sofia, Sofia, Bulgaria

**Keywords:** Anterior mediastinal thyroid tumors, Histology, Approaches, Surgical treatment, Complications

## Abstract

**Background:**

This manuscript aims to describe the symptoms, demographics, surgical approaches and techniques, the volume of surgical interventions, histological results, intra- and postoperative complications, and postoperative results in patients with anterior mediastinal tumors of thyroid origin (AMTTO).

**Methods:**

Twenty patients with AMTTO were operated between 2017 and 2021. Fifteen were women and 5 were men. The mean age was 66.8 years.

**Results:**

The most common histology was nodular micro- and macrofollicular goiter (15/20, 75%). Kocher cervicotomy (65%) was the preferred approach. Total thyroidectomy was performed in 95% of patients. Intraoperative complications were identified in 25% (5/20), and in 2 patients a tracheostomy was required. Early postoperative complications were established in 65% and the most common was unilateral transient recurrent nerve paresis or paralysis and dysphonia (25%).

**Conclusions:**

Commonly resection of AMTTO is a challenge due to its complexities associated with high-risk cases, emphasizing the need for experienced centers in managing such cases.

## Introduction

The three-compartment cross-sectional imaging model of the mediastinum includes anterior, middle, and posterior compartments [1]. Tumors of the anterior mediastinum are the most common group and they include thymic masses, lymphomas, germ-cell neoplasms, and tumors of thyroid origin. Mediastinal tumors of thyroid origin are most often secondary and they originate from the thyroid gland, descending progressively to the mediastinum from the neck.

This manuscript aims to describe the symptoms, demographics, surgical approaches and techniques, the volume of surgical interventions, histological results, intra- and postoperative complications, and postoperative results in patients with anterior mediastinal tumors of thyroid origin (AMTTO).

## Materials & methods

For the period of 5 years (2017 to 2021), 20 patients with AMTTO were operated by the same surgeon at the Thoracic Surgery Department. Chest x-ray, cervical/thoracic ultrasound, and computed tomography (CT) imaging modalities were performed. One year follow-up was conducted. The symptoms, demographics, surgical approaches, surgical techniques, the volume of surgical interventions, histological results, intra- and postoperative complications, and postoperative results were recorded.

All patients signed informed consent for all the procedures as a part of their hospital stay. Local Ethical Committee approved the study (Ethics Committee of the Department of Respiratory Diseases, number 1/16.08.2022).

## Results

The mean age was 66.8 years (34–84 years) and the majority of patients were female (15/20, 75%).

**Fig. 1:**
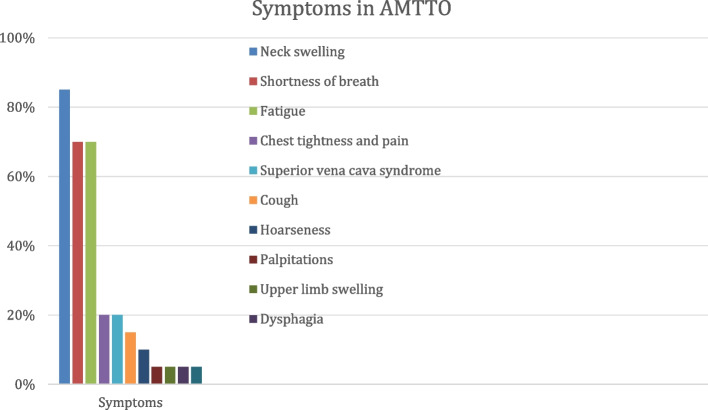
Symptoms in patients with AMTTO

AMTTO caused symptoms in 18/20 (90%) of patients. The major complaints were neck swelling (17/20, 85%), shortness of breath, and fatigue (each in 14/20, 70%) (Fig. 1). Less common symptoms include chest tightness and pain (4/20, 20%), superior vena cava syndrome (edema, collaterals of the head, neck, upper limbs, and anterior chest wall) (4/20, 20%), cough (3/20, 15%), hoarseness (2/20, 10%), palpitations (1/20, 5%), upper limb swelling (1/20, 5%), dysphagia (1/20, 5%), limb pain and pathological fracture due to primary hyperparathyroidism (1/20, 5%). In 2 patients the disease was asymptomatic and was detected on prophylactic ultrasound thyroid follow-up.

Physical examination revealed a diffusely enlarged thyroid gland with a nodular surface, painless on palpation, dense-elastic consistency, poor mobility, and mediastinal descendance. Stridor and hoarseness were present in 4 patients (20%). The majority of patients were euthyroid (19/20, 95%), and 1 patient was with hypothyroidism. It should be noted that 1 patient was with hyperparathyroidism.

The most common concomitant disease was arterial hypertension (14/20, 70%). In 5 patients (25%) a recurrent disease was diagnosed after previous surgery: 3—nodular goiter; 1—adenoma of a parathyroid gland; 1—paraganglioma. In 2 patients (10%) a synchronous tumor was detected (1—carcinoma of the left auricle; 1—breast cancer).

**Fig. 2:**
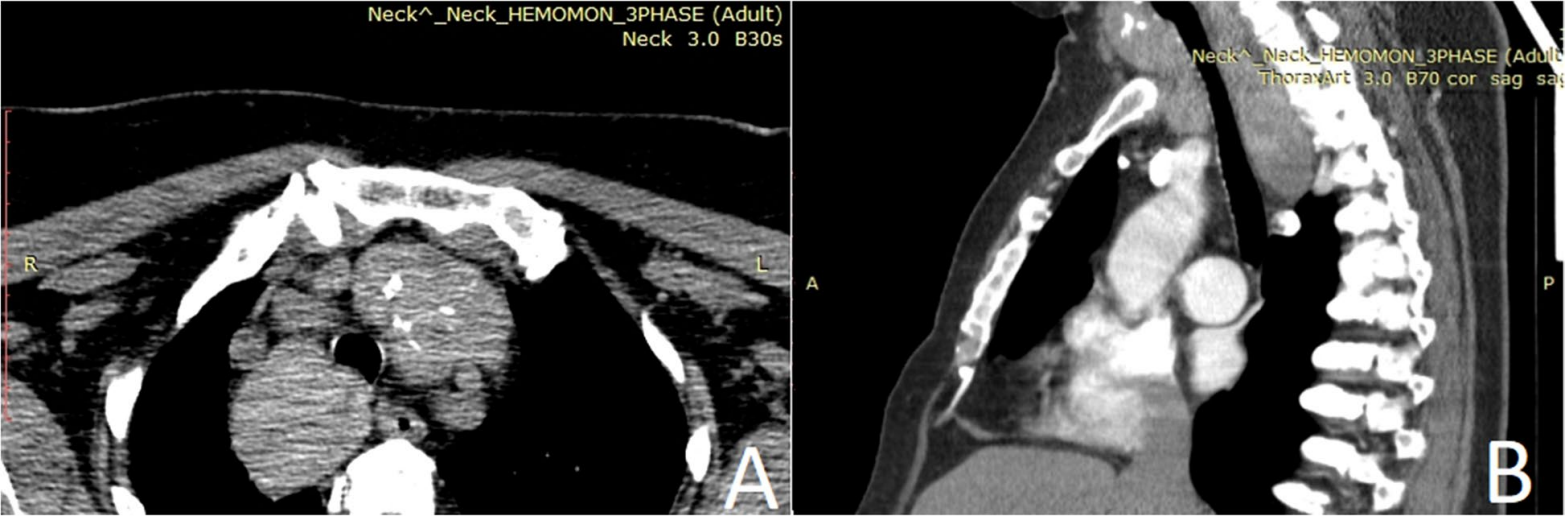
CT scan revealing a retrosternal goiter. **A** Axial image. **B** Sagittal image

CT was performed in all 20 patients (100%). The lesions were presented as heterodense masses with hypodense nodules, calcifications, and cystic areas, and after intravenous contrast administration, they increased their density inhomogeneously (Fig. 2). Cervical and/or thoracic ultrasound found nodular goiter with intrathoracic propagation, iso- and hypoechoic nodules, microcalcifications, cystic lesions, heteroechoic formations, vascular compression and thrombosis, and pathological cervical lymphadenomegaly.

The formation engages both lobes, but predominantly the right lobe in 8 patients (40%) and the left lobe in 3 patients (15%). In 4 patients (20%) its origin was entirely from the left lobe, and in 2 patients (10%)—from the right lobe. Only one case had a true mediastinal recurrent paraganglioma. Tracheal compression or stenosis on CT scan was present in 12 patients (60%) with a minimum diameter of 3 mm and was combined with esophageal compression in 4 patients. It affected the adjacent vascular structures and caused innominate vein stenosis or compression (9/20, 45%), jugular vein compression (3/20, 15%), subclavian vein compression (1/20, 5%), aortic arch compression (1/20, 5%), common carotid artery compression (1/20, 5%), subclavian artery thrombosis (1/20, 5%), subclavian vein thrombosis (1/20, 5%), and jugular vein dilatation (1/20, 5%).

Preoperative fiberoptic bronchoscopy was performed in all 20 patients (100%) and it found tracheal compressive narrowing or stenosis (10/20, 50%), erased surface of tracheal rings (8/20, 40%), paresis of one vocal cord (3/20, 15%), of two vocal cords, tracheal deviation, edematous vocal cords (1 patient each, 5%). Vocal cord paresis was defined as decreased but perceptible motion and paralysis implied vocal fold immobility.

**Fig. 3:**
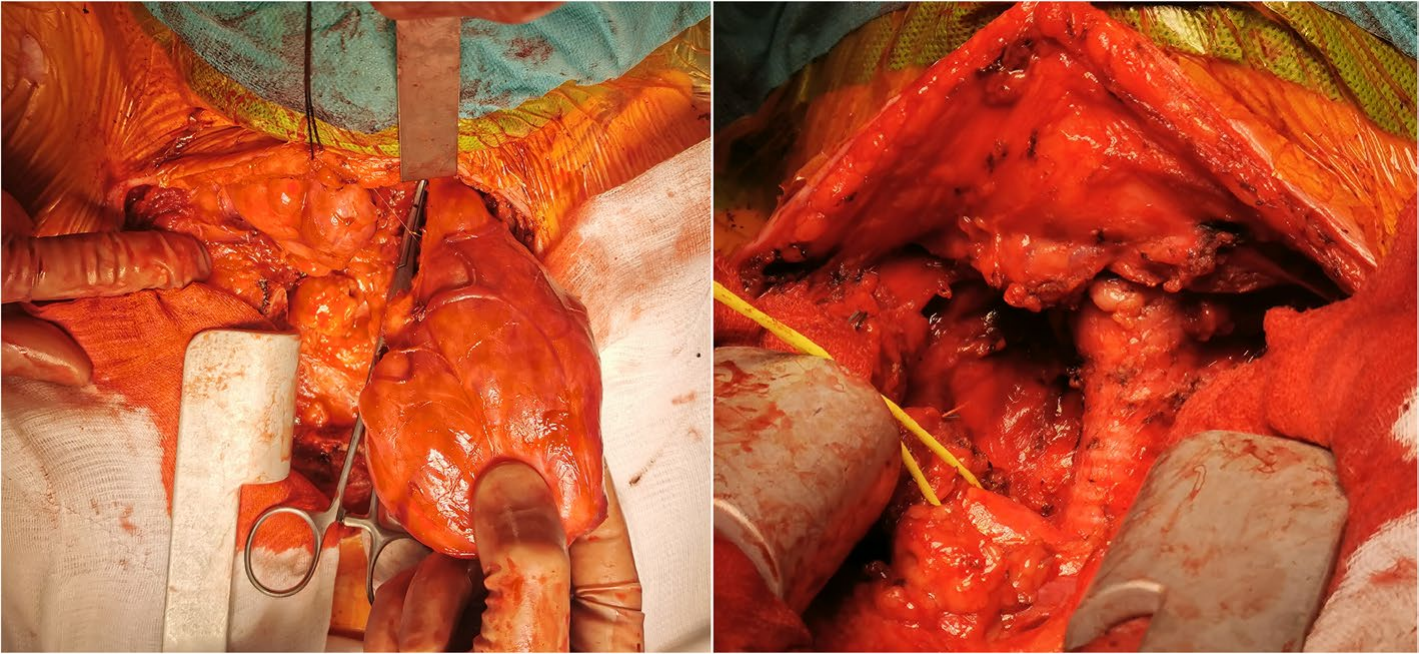
Intraoperative images. **A** Collar neck incision and proximal partial sternotomy with the left thyroid lobe mobilized. **B** A view after performing total thyroidectomy for nodular goiter (truncus brachiocephalicus was hanged on a tourniquet)

Sixty-five percent of patients were operated by cervicotomy (13/20). The other approaches were cervicotomy + partial sternotomy (2 patients, 10%), cervicotomy + total sternotomy (2 patients, 10%), cervicotomy + lateral/anterolateral minithoracotomy (2 patients, 10%), or only resternotomy (1 patient, 5%) (Fig. 3). In 19 patients (95%) a total thyroidectomy was performed, and in 1 patient (5%)—unilateral subtotal lobectomy. Additional surgical procedures were: tracheostomy (2 patients, 10%), segmental tracheal resection and plasty (1 patient, 5%), thymectomy (1 patient, 5%); resection of a main venous vessel (2 patients, 10%—brachiocephalic vein and internal jugular vein – 1 patient; resection of brachiocephalic vein – 1 patient).

**Fig. 4:**
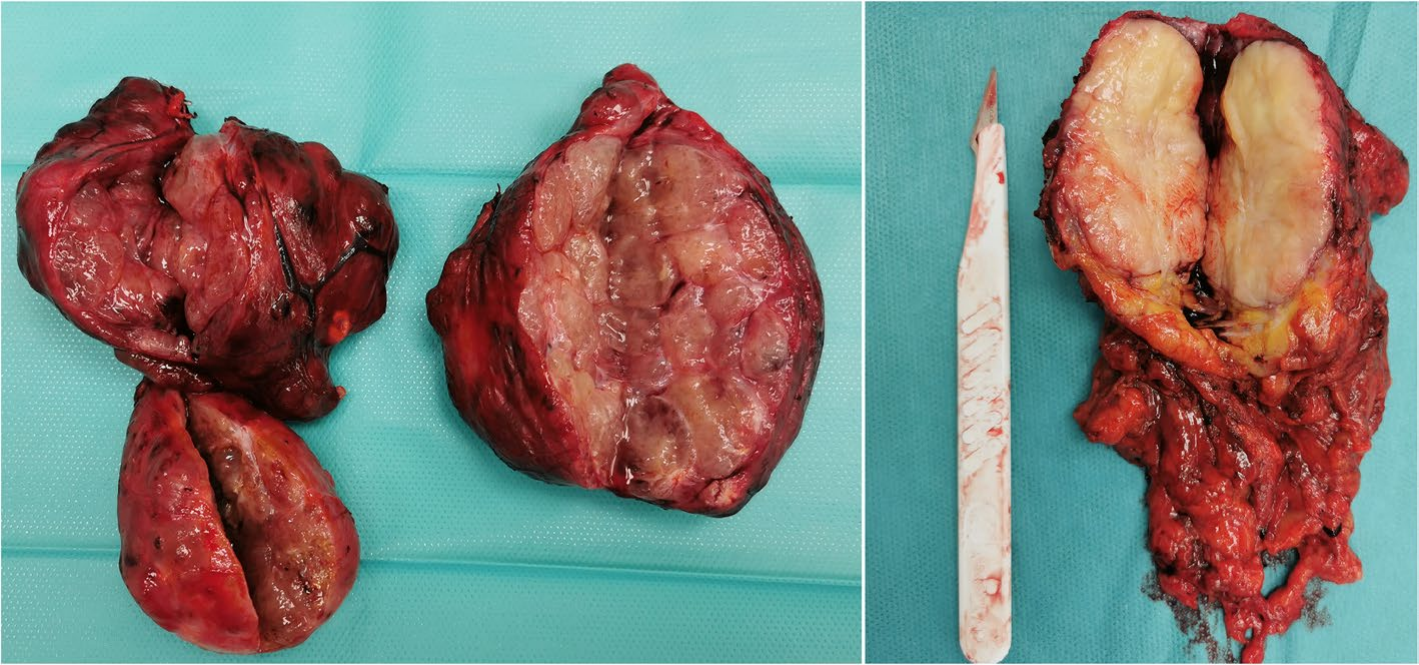
Postoperative specimens. **A** Retrosternal nodular goiter—right and left lobes. **B** Recurrent malignant mediastinal paraganglioma of thyroid origin

The main part of AMTTO histology was benign, as nodular micro- and macrofollicular goiter (15/20, 75%) was the most common histology. Other less common causes were: invasive fibrous thyroiditis (Riedel's thyroiditis) (2 patients), follicular adenoma (2 patients), combined anaplastic + papillary thyroid carcinoma, papillary carcinoma, low-grade thyroid carcinoma, thyroid paraganglioma and recurrent paraganglioma of thyroid origin, recurrent parathyroid adenoma (with dimensions of 14/7 mm in diameter), and follicular microfollicular mixed with fetal adenoma (1 patient each) (Fig. 4). In addition, from all 15 patients with goiter 11 were resected only through neck collar incision.

All patients underwent R0 resection except the patient with low-differentiated carcinoma (1/20, 5%), which had macroscopically positive resection lines and a protective tracheostomy was performed.

Additional surgical procedures were: tracheostomy (2 patients, 10%), segmental tracheal resection and plasty (1 patient, 5%), thymectomy (1 patient, 5%); resection of a main venous vessel (2 patients, 10%—brachiocephalic vein and internal jugular vein – 1 patient; resection of brachiocephalic vein – 1 patient).

**Fig. 5:**
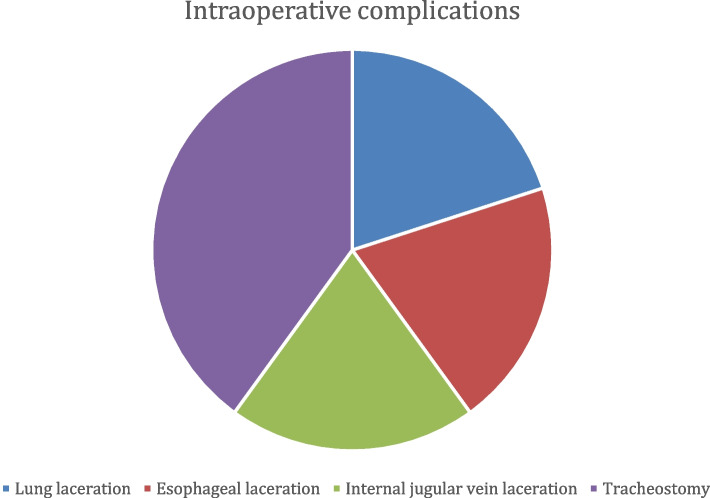
Intraoperative complications

**Fig. 6:**
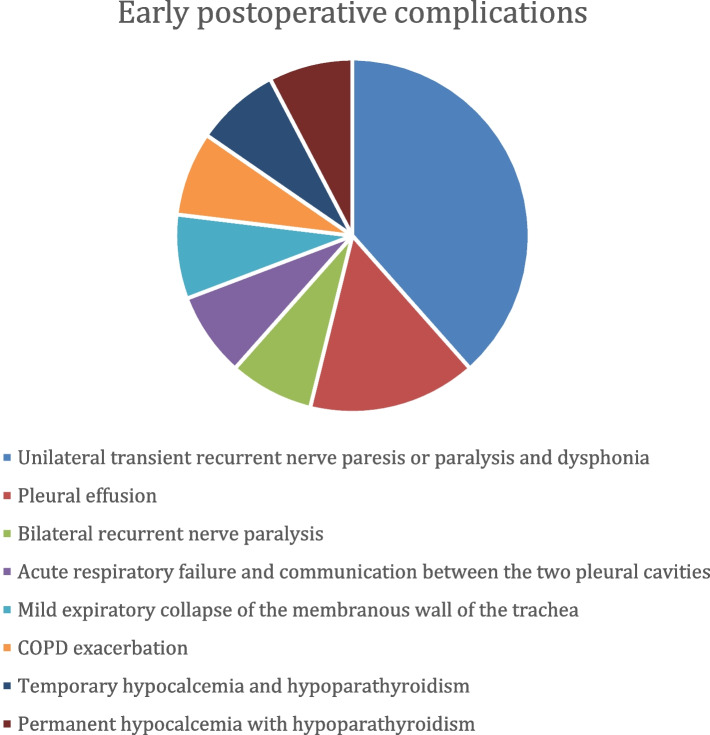
Early postoperative complications

Intraoperative complications were present in 25% of patients (5/20) – 1—small laceration of the lung, 1—mild laceration of the esophageal wall musculature, 1—small lesion of the internal jugular vein; in 2 patients (10%) a tracheostomy was required due to tracheal infiltration and bilateral recurrent laryngeal nerve injury and subsequent respiratory failure (Fig. 5). Postoperative complications were divided into two groups: early- (within 30 days) and late-onset (more than 30 days). Early postoperative complications were present in 65% (13/20): 5—unilateral transient recurrent nerve paresis or paralysis and dysphonia (25%), 2 – pleural effusion (10%), 1—bilateral recurrent nerve paralysis (5%) (this case is one of the two who underwent tracheostomy), 1—acute respiratory failure and communication between the two pleural cavities (5%), 1—a mild expiratory collapse of the membranous wall of the trachea (5%), 1—COPD exacerbation (5%), 1 – temporary, and 1—permanent hypocalcemia and hypoparathyroidism (5% each) (Fig. 6). The 5 patients with unilateral recurrent nerve paresis or paralysis were evaluated postoperatively for persistence or resolution of vocal cord weakness with the help of flexible laryngoscopy, which revealed full restoration of function. Only the patient with bilateral RLN paralysis and tracheostomy had persistent RLN damage but due to its advanced oncologic stage medialization or thyroplasty procedures were not indicated. This patient had damaged vocal cord function and bilateral paresis before the surgery. There were no late postoperative complications.

The mean hospital stay was 11 days (3–35 days). In a one-year follow-up, 19 patients were alive and in good overall condition.

## Discussion

This manuscript describes our 5-year experience with AMTTO.

The majority of mediastinal goiters are diagnosed in the sixth decade of life with a female-to-male ratio of 3:1 [2]. The mean age of patients in our study was 66.8 years and women were more affected with a female/male ratio of 3:1 which confirms the previous findings.

The most common symptoms in patients with intrathoracic goiter are cervical mass (45.8%), dyspnea and coughing (9.5%), hyperthyroidism (7.6%), dysphonia (3%), dysphagia (2.6%), emergency surgery for acute respiratory failure due to tracheal obstruction (1.9%), vein compression (1.5%) [3]. Uncommon presentation could be esophageal varices with bleeding [4], superior vena cava syndrome, cerebral edema, or transient ischemic events [5]. The so-called ‘downhill’ esophageal varices in AMTTO could develop due to superior vena cava syndrome and functionate as collaterals to bypass superior vena cava occlusion via the azygos vein or to drain the superior system to the portal vein in case of both the superior vena cava and the azygos vein are blocked [4]. In our study, AMTTO caused symptoms in 90% of patients. We identified neck swelling (85%) and shortness of breath and fatigue (70%) as the most common symptoms.

CT is the gold standard in preoperative diagnosis, evaluation, and staging of AMTTO. Most intrathoracic goiters present in the right mediastinum as thyroidal appendages and expansion in the left mediastinum is hampered by the thymus gland and the great vessels of the middle mediastinum [4]. In our study, the origin was predominantly from the two lobes of the thyroid gland (55%) and the right lobe was affected more often than the left one.

A heterogeneous prevascular mediastinal mass that demonstrates continuity with the cervical thyroid gland, is intrinsically hyperattenuating (with Hounsfield unit values of 70–85 due to the presence of iodine), and demonstrates intense and sustained enhancement after intravenous contrast medium points towards a mediastinal goiter [1]. Scintigraphy with Tc-99 m, I-123, or I-131 is the most important diagnostic tool to detect extrathoracic thyroid gland [5].

Surgery is indicated in all AMTTO due to the risk of progressive enlarging in size with consequent compressive symptoms, malignant degeneration, and the need for histological diagnosis. Tracheal compression causing dyspnea was one important factor for surgery irrespective of the level. The tracheal stenosis was quantified by fiberoptic bronchoscopic examination and CT scan. Of note are the two cases with Riedel’s thyroiditis. Usually, the treatment of Riedel’s thyroiditis includes steroids, immune modulators, tamoxifen, etc. Surgery in this setting is reserved for steroid-resistant cases, severe adverse effects of medical therapy, suspicion of malignancy, and for relieving obstructive symptoms. In the first case, surgery was performed due to the significant tracheal compression, and CT data indicating malignancy and negative preoperative histological diagnosis by transcutaneous fine-needle biopsy. In the second case, the surgery was performed due to dyspnea because of critical endoluminal tracheal stenosis and an inconclusive preoperative endoscopic biopsy for malignant disease.

Surgical approaches include cervicotomy *a modo* Kocher, thoracotomy, sternotomy, and video-assisted and robotic-assisted surgeries. Our team do conventionally cervicotomy as an 8- to 10-cm curvilinear incision one finger breadth above the sternal notch. In this study, the preferred approach in 65% of patients with AMTTO is also Kocher cervicotomy only. Some of the described techniques to mobilize substernal thyroids out into the neck are the following: commence with mobilization of the smaller lobe; preservation of parathyroid glands, especially the superior ones; division of the strap muscles; isthmotomy; separately extirpation of the two lobes; mobilization of the upper thyroid lobes and traction of the gland towards the head [6]. However, a sternotomy is indicated when a lesion is large, descends deeply in the mediastinum, or is with retrovascular location. Very often these lesions are long-standing and thus, have dense adhesions with the surrounding main blood vessels and airways, recurrent nerves, trachea, and esophagus, which implies a great risk of complications with cervicotomy only. Maneuvers involving blind dislocation of the gland from the mediastinum towards the neck (e.g., Foley catheter applied through the cervical incision) are discouraged due to the high risk of hemorrhage or damage to adjacent structures located in the thoracic inlet [2]. A thyroid volume of ≥ 162 cm^3^ extending below the thoracic inlet is a significant determining factor for an extra-cervical approach [7]. Unfortunately, we have not measured for all target volumes. Other indications of median sternotomy are a goiter extended below the aortic arch or tracheal bifurcation, and mediastinal ectopic thyroid tissue [8]. One study reported the Kocher incision as the most common surgical approach (96.6%), while an accessory incision was performed only in 3.4% [3]. Thirteen patients in our study were operated by Kocher cervicotomy (65%). Extracervical approach was used in 30% of this patient group in a preplanned fashion—in all patients with adding total sternotomy/thoracotomy to cervicotomy, in the resternotomy case, and in 1 patient with cervicotomy + partial proximal sternotomy. In the other patient with cervicotomy + partial proximal sternotomy extracervical approach (5%) was used after the neck approach was unsuccessful. All planned extracervical approaches were executed and no one was cancelled. In these cases, it was impossible to perform the surgery only through the neck collar incision. The higher rate of preplanned extracervical approach could be explained with that as the leading clinic for thoracic surgery most of our patients were complicated. In addition, robotic-assisted procedures were not used, because of Da Vinci Robotic Surgical System is not available in our clinic. The thoracoscopic approach was very risky in this patient population due to the malignant histology, anatomic localization, adjacent infiltration, higher bleeding risk and therefore not applicable. According to our study, we found some factors with elevated risk for extra-cervical approach. From all 7 patients with extra-cervical approach superior vena cava syndrome was present in 2 patients in comparison to 2 cases in the cervicotomy group. From 7 patients with extra-cervical approach malignant histologic results were proved in 4 patients in comparison to one 1 case in the cervicotomy group. In all 7 patients with extra-cervical approach, there was a descendance to aortic arch or tracheal bifurcation compared to only 8 cases in the cervicotomy group (Table 1).

**Table 1:** Factors, associated with elevated risk for extra-cervical approach in AMTTO

	Cervicotomy only (13 patients)	Extra-cervical approach (7 patients)
Superior vena cava syndrome	2 (15.38%)	2 (28.57%)
Malignant histology	1 (7.69%)	4 (57.14%)
Descendance to aortic arch/tracheal bifurcation	8 (61.54%)	7 (100%)

According to one study, 58.5% of the retrosternal goiters were multi-nodular goiter, 22.9% were papillary cell carcinoma, 7.1% were medullary carcinoma, 5.7% were combined anaplastic + papillary carcinoma, 5.7% were thyroid lymphoma, and only 1.4% were thyroid adenoma [9]. In our study, the main part of AMTTO histology was benign, as nodular micro- and macrofollicular goiter (15/20, 75%) was the most common histology. Other histologic types were: invasive fibrous thyroiditis (10%), follicular adenoma (10%), combined anaplastic + papillary thyroid carcinoma, papillary carcinoma, low-grade thyroid carcinoma, paraganglioma and recurrent paraganglioma, recurrent parathyroid adenoma, and microfollicular partial fetal adenoma (5% each). The mediastinal parathyroid adenoma was included in this article because of its closely related embryogenesis and anatomical proximity to the thyroid gland. Moreover, the patient had also a synchronous goiter.

Some of the reported postoperative complications are tracheal collapse, RLN injury, prolonged mechanical ventilation, secondary hypoparathyroidism/hypocalcemia, hemorrhage, and wound infection. Tracheomalacia presents with low incidence (0–2%) after resection of substernal goiters [2]. Other authors stated, that transient hypocalcemia occurs in 11.9% [7]. Rates of permanent RLN injury following substernal goiter surgery are 2–4% [8]. In our study, the intraoperative complications were present in 25% of patients (5/20) and the most common early postoperative complications (65%) were unilateral transient recurrent nerve paresis or paralysis and dysphonia (5/20, 25%). Our team used RLN identification as the well-accepted gold standard during thyroidectomy. There are four different surgical approaches (lateral, inferior, superior, and medial) that help in intraoperative identification of RLN [10]. To note, the anatomical position of the nerve is commonly distorted, it must be identified at the cricothyroid junction or near the inferior thyroid artery, and careful management of Berry’s ligament is necessary, because of its proximity to the RLN [6]. Laryngeal electromyography (EMG) monitoring during surgery was not used by our team, because its effectiveness in AMTTO is controversial and our facility lacks the relevant equipment. Moreover, some articles stated that presently there is no conclusive data for the superiority or inferiority of IONM over intraoperative RLN visualization only for the prevention of RLN damage [11]. We attribute the relatively high complication rate to the large dimensions of the tumors, long-standing disease history, mediastinal tumor position, and the predominantly used cervical approach, which hinders the view of dissection.

Non-surgical therapy with radioactive iodine ablation should be reserved only where general anesthesia is inappropriate because it can also result in an acute inflammatory process that exacerbates clinical compression, potentially threatening the patient’s airway [8].

We are obligated to mention some limitations of this study, for instance, the small patient population, consisting of 20 patients. Moreover, as a highly-specialized center for thoracic surgery diseases, most of the patients referred to our clinic are complicated cases, with recurrent diseases, often with aggressive and advanced cancers. Therefore, this may produce some bias in our results. For example, in our study, there is a very high rate of malignancy of the thyroid, including relatively uncommon variants. We found also only 75% of the patients have a multinodular benign goiter in comparison to the results from a large series of substernal thyroid masses from developed countries, where its rate is > 90%. Despite this, we would like to emphasize the high medical significance of our study, as it encompasses a heterogenous group of AMTTO, which was not described elsewhere in the medical literature till now according to our data. Our study serves as inspiration stimulating more centers to create a large multicentric prospective study not only for AMTTO but for thyroid tumors of all mediastinal compartments.

## Conclusions

Surgery is the method of first choice in all patients with AMTTO due to the risk of progressive increase in tumor size with subsequent compression symptoms, malignant degeneration, and histological verification. Cervicotomy was the most preferred approach and nodular micro- and macrofollicular goiter was the most common histology. Commonly resection of AMTTO is a challenge due to its complexities associated with high-risk cases, emphasizing the need for experienced centers in managing such cases.

## Data Availability

The datasets used and/or analysed during the current study are available from the corresponding author on reasonable request.
